# Effects of *Bacillus subtilis* CSL2 on the composition and functional diversity of the faecal microbiota of broiler chickens challenged with *Salmonella* Gallinarum

**DOI:** 10.1186/s40104-016-0130-8

**Published:** 2017-01-05

**Authors:** Ju Kyoung Oh, Edward Alain B. Pajarillo, Jong Pyo Chae, In Ho Kim, Dong Soo Yang, Dae-Kyung Kang

**Affiliations:** 1 Department of Animal Resources Science, Dankook University, 119 Dandae-ro, Cheonan, 31116 Republic of Korea; 2 Abson BioChem, Inc, 10-1 Yangjimaeul-gil, Sangrok-gu, Ansan, 15524 Republic of Korea

**Keywords:** *Bacillus subtilis*, Broiler chicken, Microbiome, *Salmonella* Gallinarum, 16S rRNA gene

## Abstract

**Background:**

The chicken gastrointestinal tract contains a diverse microbiota whose composition and structure play important roles in gut functionality. In this study, microbial shifts resulting from feed supplementation with *Bacillus subtilis* CSL2 were evaluated in broilers challenged and unchallenged with *Salmonella* Gallinarum. To analyse bacterial community composition and functionality, 454 GS-FLX pyrosequencing of 16S rRNA gene amplicons was performed.

**Results:**

The Quantitative Insights into Microbial Ecology (QIIME) pipeline was used to analyse changes in the faecal microbiota over a 24-h period. A total of 718,204 sequences from broiler chickens were recorded and analysed. At the phylum level, Firmicutes, Bacteroidetes, and Proteobacteria were the predominant bacterial taxa. In *Salmonella*-infected chickens (SC), Bacteroidetes were more highly abundant compared to control (NC) and *Bacillus*-treated (BT) chickens. At the genus level, in the NC and BT groups, *Lactobacillus* was present at high abundance, and the abundance of *Turicibacter*, unclassified *Enterobacteriaceae*, and *Bacteroides* increased in SC broilers. Furthermore, taxon-independent analysis showed that the SC and BT groups were compositionally distinct at the end of the 24-h period. Further analysis of functional properties showed that *B. subtilis* CSL2 administration increased gut-associated energy supply mechanisms (i.e. carbohydrate transport and metabolism) to maintain a stable microbiota and protect gut integrity.

**Conclusions:**

This study demonstrated that *S.* Gallinarum infection and *B. subtilis* CSL2 supplementation in the diet of broiler chickens influenced the diversity, composition, and functional diversity of the faecal microbiota. Moreover, the findings offer significant insights to understand potential mechanisms of *Salmonella* infection and the mode of action of probiotics in broiler chickens.

**Electronic supplementary material:**

The online version of this article (doi:10.1186/s40104-016-0130-8) contains supplementary material, which is available to authorized users.

## Background

Poultry is one of the most important meat sources for humans [1]. Due to the increasing demand for food, chicken production has increased tremendously in the past few years [1]. Therefore, husbandry and management are vital for preventing infection and maintaining the health of poultry. Animal health is closely associated with the status of the gastrointestinal tract, whose disruption or dysbiosis leads to detrimental effects [2]. Intestinal homeostasis and functionality are influenced by various factors, such as (1) diet and feed additives, (2) farm conditions and practices, and (3) the resident gut microbiota [3]. The microbiota comprises trillions of microorganisms localised primarily at the distal end of the gastrointestinal tract [4]. These microbial communities mediate digestion of feedstuff, control gut homeostasis, and prevent infection.

*Salmonella enterica* is an important group of gastrointestinal pathogens that causes food-borne diseases, gastroenteritis, and diarrhoea in animals and humans [2, 5, 6]. Several serovars of *S. enterica* subsp. *enterica*, specifically *S.* Enteritidis and *S.* Gallinarum, are major avian pathogens. These serovars are frequently associated with poultry salmonellosis, which results in severe morbidity and mortality [6]. The pathogenicity and routes of transmission of *Salmonella* spp. have been investigated extensively; however, few studies have addressed *S.* Gallinarum infection and its control in poultry [6].

Probiotics are live microorganisms that confer a wide range of benefits on animals, such as stimulation of immune responses, maintenance of gut barrier function, and prevention of pathogen invasion of the gut [1, 7] The gram-positive bacterium *Bacillus subtilis* has been used as an in-feed probiotic supplement for livestock and poultry [8, 9]. Only recently have studies begun to address the effects of probiotic interventions on the gastrointestinal microbiota. However, previous studies using plating methods, 16S rRNA gene clone libraries, denaturing gradient gel electrophoresis, and so forth have yielded relatively little information [9]. In contrast, high-throughput next-generation sequencing (NGS) methods facilitate rapid quantification and identification of bacterial communities [4, 10]. In addition, simultaneous analysis of multiple samples enables a thorough understanding of microbial communities and therefore prediction of the effect of interventions and infections on microbial functions and community diversity [11]. This study applied 16S rRNA gene sequencing to investigate the composition of the chicken gut microbiota, and the findings suggest a marked effect of *S.* Gallinarum on the overall composition and metabolic functions of the chicken gut microbiota. In addition, the data suggest that *B. subtilis* CSL2 exerts protective effects against *S*. Gallinarum infection by altering the faecal microbiota of broiler chickens.

## Methods

### Animals and experimental design

A total of 36 Ross-308 broiler chickens were bred and divided into the following three groups: the control group (NC; *n* = 12), *Salmonella*-challenged (SC; *n* = 12) group, and *Bacillus*-treated (BT; *n* = 12) group. From day 1, the NC and SC groups were given the standard basal diet (Additional file 1: Table S1), while the BT group was fed a probiotic-supplemented basal diet containing *Bacillus subtilis* CSL2 (GenBank accession number: KX281166) at 1.0 × 10^7^ colony forming units (CFU)/g feed. Freeze-dried *B. subtilis* CSL2 (1.0 × 10^10^ CFU/g) diluted with basal diet by mixing for 2 h with a feed mixer (Daedong Tech, Korea), to obtain final concentration of 1.0 × 10^7^ CFU/g feed. Feeding of the respective diets continued until d 31 (before). On d 32, chickens in the SC and BT groups were orally challenged with *S. enterica* subsp. Gallinarum KVCC-BA0700722 (*S.* Gallinarum) at 1.0 × 10^8^ CFU/mL. The Dankook University Animal Care Committee approved all animal protocols.

All 36 chickens (12 birds per treatment) were tagged and placed in cages randomly, which were equipped with a nipple water dispenser for ad libitum access to water, together with a one-sided self-feeder. The room temperature was maintained at 32 °C for the first week, and then reduced 3 °C weekly until the temperature reached 26 °C. The broiler chickens received no antibiotics or other additives during the study period. No additional chickens were introduced during the duration of the experiment. At 31 and 32 d, 12 chickens per treatment were selected for faecal sampling. Fresh faecal samples were collected aseptically from the rectum of the broiler chickens (on d 31: before; on d 33: after). Finally, faecal contents were placed into sterile tubes and kept on ice until used for microbiota analysis on the same day of the sampling.

### Sample preparation and DNA isolation

Genomic DNA isolation from freshly collected faeces was carried out using a technique described previously [11]. Briefly, 0.3 g of faecal extract was placed in a bead-beating tube containing garnet beads. Lysis of host and microbial cells was mediated by both mechanical collisions between beads and chemical disruption of cell membranes. DNA was purified using a Power Faecal® DNA Extraction Kit (MO BIO Laboratories, Inc., USA) as per the manufacturer’s instructions. Precipitated DNA was suspended in DNase-free H_2_O, and its concentration and purity were assessed by UV/vis spectrophotometry and agarose gel electrophoresis, respectively (Mecasys Co., Ltd, Korea).

### 454-Pyrosequencing analysis

DNA amplicons from individual broiler chicken samples were amplified using primers for the V1-V3 hyper-variable regions of the 16S rRNA gene by polymerase chain reaction (PCR). Forward primers were tagged with 10-bp unique barcode labels at the 5’ end along with the adaptor sequence to allow multiple samples to be included in a single 454 GS FLX Titanium sequencing plate, as described previously [11]. Finally, 16S rRNA amplicons were quantified, pooled, and purified for sequencing.

### Data processing

The 16S rRNA sequence data generated by the 454 GS FLX Titanium chemistry (Roche) were processed using the Quantitative Insights into Microbial Ecology (QIIME) pipeline. Briefly, sequences that were less than 200 bp or greater than 600 bp in length, were of low quality, contained incorrect primer sequences, and/or contained more than one ambiguous base were filtered using the split_libraries.py script. After checking for chimeric sequences, sequence data were filtered using the identify_chimeric_seqs.py and filter_fasta.py scripts. Operational taxonomic units (OTU) were picked using the pick_open_reference.py script and the most recent Greengenes reference database (13_8) at a 97% identity threshold. Bacterial composition data from broiler faecal samples were generated using the summarize_taxa_through_plots.py scripts. For alpha diversity measurements, the alpha_diversity.py script was employed to generate Chao1, Shannon, Simpson, and phylogenetic distance (PD) whole-tree values. Rarefaction curves were also generated in the QIIME software. All reads were pooled for each group of broiler chickens. Sequences were rarefied according to sequencing depth to visualise the change in diversity with respect to sampling depth.

### Statistical analysis

Statistical and multivariate analyses were performed using the R software (v. 3.1.0; R Core Team, Auckland, New Zealand). The proportions of bacterial taxa (phylum and genus level) were compared between groups before and after the 24 h challenge. To avoid statistical bias and perform valid downstream analysis, the normalisation of OTU table was performed using the base package in R software. For multivariate analysis of bacterial OTU at a 95% identity threshold, the adegenet package in the R software was used to determine the peaks of the bacterial genera that facilitated discrimination according to the defined clustering groups with a user-defined threshold in the canonical loading plot. The NC, SC, and BT groups were labelled accordingly. In discriminant analysis of principal components (DAPC) (dapc {adegenet}), the normalised abundance data of individual samples was employed [12]. The number of principal components was ≥80% of the cumulative variance explained by the eigenvalues of the DAPC plot, and these principal components were subjected to linear discriminant analysis (LDA), resulting in selection of ≥2 linear discriminants for the DAPC plot [12]. The visual outputs of the canonical loading plot and the DAPC plot were then created using (loadingplot {adegenet}) and scatter plot (scatter {ade4}), respectively. Furthermore, functional prediction was carried out using the Phylogenetic Investigation of Communities by Reconstruction of Unobserved States (PICRUSt) based on the Greengenes 16S rRNA database and KEGG Orthologs (KO) [10]. PICRUSt was used to identify differences in the functional potential of bacterial communities among the groups. Using KEGG (level 3) ortholog function predictions, differences among NC, SC, and BT groups were evaluated, and a loading plot was created to identify the most discriminating functions among the groups after 24 h. Tukey’s honestly significant difference (HSD) test was employed to evaluate functional differences among the groups; a *P*-value <0.05 was considered to indicate significance.

## Results

### DNA sequence data and quality control

Pyrosequencing analysis generated a total of 718,204 raw sequence reads. The average number of reads per sample was 10,941, and the mean number of sequence reads per group ranged from 7,701 to 14,199 (Table 1). The average number of reads per sample is comparable to previous animal studies that utilised the GS FLX Titanium system [13, 14]. Barcoded primers allowed pooling of samples for individual- and group-based analyses. At a 95% identity cut-off (genus level), 212 unique operational taxonomic units (OTU) were detected in this study using the latest Greengenes database (13_8); these were used for downstream analyses. The recorded mean OTU per group ranged from 421 to 725 (Table 1).

**Table 1:** Pyrosequencing data and diversity indices of the faecal microbiota of broiler chickens

Group*	Diversity indices (Mean ± standard deviation)					
	No. of reads^a^	OTU	Chao1	Shannon	Simpson	PD
NC (Before)	12,532 ± 6,666	725 ± 420	1,443 ± 752	5.41 ± 1.19	0.92 ± 0.05	48.8 ± 25.75
NC (After)	10824 ± 3,322	712 ± 369	1,555 ± 729	5.79 ± 1.20	0.94 ± 0.04	48.8 ± 21.71
SC (Before)	12,176 ± 8,221	693 ± 447	1,418 ± 938	5.50 ± 0.85	0.93 ± 0.04	45.4 ± 28.49
SC (After)	7701 ± 3,665	421 ± 240	890 ± 482	4.61 ± 1.00	0.85 ± 0.07	30.7 ± 15.04
BT (Before)	8212 ± 4,494	493 ± 274	1,023 ± 574	4.98 ± 1.13	0.88 ± 0.07	36.5 ± 20.07
BT (After)	14,199 ± 5,013	580 ± 254	1,047 ± 360	5.06 ± 1.03	0.90 ± 0.06	36.8 ± 14.35

*Legend: *NC, *negative control; *SC, Salmonella* challenged; *BT, Bacillus* treated; *OTU*, operational taxonomic unit; *PD,* phylogenetic distance (whole tree)

^a^Mean number of raw reads per treatment group. No significant differences (*P* < 0.05) in alpha diversity were detected between groups using compare_alpha_diversity.py script

### Microbial diversity

Alpha diversity was compared among the NC, SC, and BT groups (Table 1; Additional file 2: Fig. S1). α-diversity parameters were calculated based on the OTU using the phylogenetic diversity (PD) whole tree, Chao1, Shannon, and Simpson methods. Diversity (Shannon and Simpson) values were highest in the NC group and lowest in SC broilers after *Salmonella* challenge (Additional file 2: Fig. S1A). Diversity values are summarised in Table 1. Species richness (Chao1) and bacterial diversity (Shannon and Simpson) exhibited similar trends; i.e. the highest values of both were in the NC group, and lowest in the SC group (Table 1), implying that *Salmonella* infection negatively affects overall microbial diversity. In addition, the rarefaction curves confirmed that *Salmonella* infection decreased the level of microbial diversity (Additional file 2: Fig. S1B).

### Effect of *Salmonella* challenge and *B. subtilis* CLS2 administration on microbiota composition

**Fig. 1:**
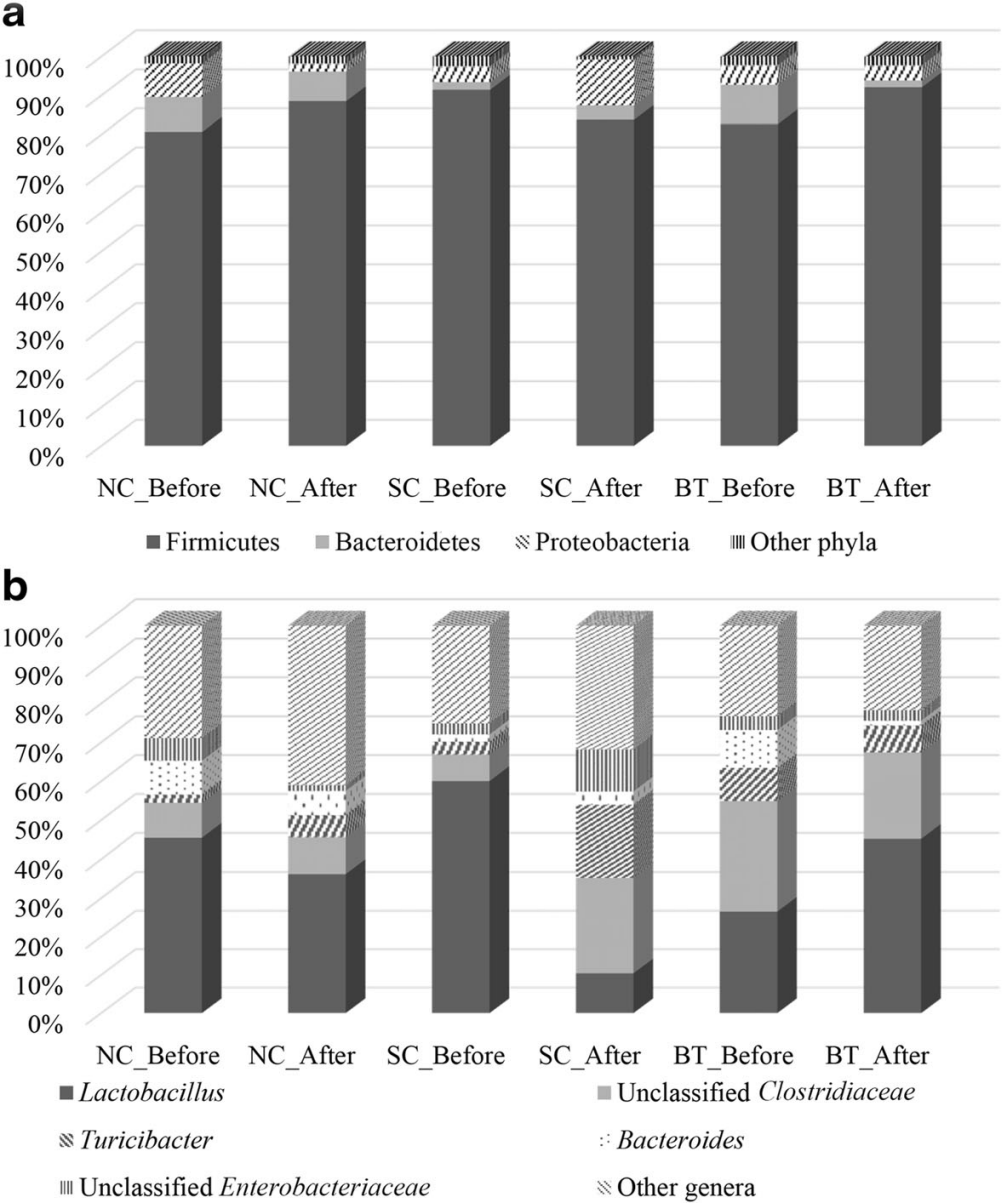
Faecal microbiota composition of broiler chickens at the phylum (**a**) and genus (**b**) levels. Broiler chickens were divided into the following three groups before and after *Salmonella* challenge: NC, control/basal diet; SC, basal diet challenged with *S.* Gallinarum (SC); and BT, basal diet supplemented with *B. subtilis* CSL2

Irrespective of *Salmonella* challenge and *Bacillus* supplementation, the phylum Firmicutes showed the highest abundance (>80%), followed by the phyla Bacteroidetes and Proteobacteria (Fig. 1a). These major bacterial phyla are also major constituents of the gut microbiota of other birds and livestock animals [13]. At the 24-h post-*Salmonella* challenge, the abundance of Proteobacteria was significantly increased, and that of Firmicutes decreased in the SC group, whereas those in the BT group had recovered to levels similar to the NC group. In previous reports, Proteobacteria was highly abundant in *Salmonella*-infected animals [15, 16]. Similarly, dietary supplementation with probiotics altered the microbial composition of broiler chickens, resulting in higher abundance of Firmicutes and lower abundance of Bacteroidetes and Proteobacteria [14].

At the genus level, a total of 124 bacterial genera were detected in all broiler samples (Additional file 3: Table S2), which is comparable to other avians [17]. *Lactobacillus*, unclassified *Clostridiaceae*, *Turicibacter*, *Bacteroides*, and unclassified *Enterobacteriaceae* were the major bacterial genera in broiler faeces (Fig. 1b). Interestingly, the abundance of *Lactobacillus*, a genus of beneficial bacteria, decreased significantly in the SC group, but increased in the BT group. In addition, the abundance of unclassified *Enterobacteriaceae*, which comprises several pathogenic species, in the SC group increased from 2.9% to 10.9%. A previous study reported an increase in *Lactobacillus* abundance after probiotic administration in chicken and pigs [18]. In contrast, SC broilers had a higher abundance of *Turicibacter*, unclassified *Enterobacteriaceae*, and *Bacteroides* than the other groups had. In a previous study, *Salmonella* infection of mice was also found to result in higher *Enterobacteriaceae* and lower *Lactobacillus* abundance [19].

### Taxon-independent and functional analyses

**Fig. 2:**
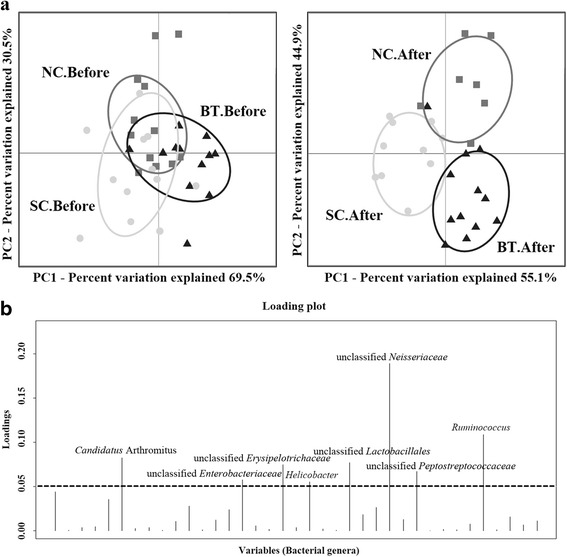
Taxon-independent multivariate analysis and separation of broiler microbiota. **a** Discriminant analysis of principal components revealed distinct clustering of the control (NC, *grey*), *Salmonella*-challenged (SC, *white*), and *Bacillus*-treated (BT, *black*) groups using OTU at the 97% identity level. Significant differences (*P* < 0.001) were calculated using compare_categories.py using the PERMANOVA test. **b** Canonical loading plot showing differentially abundant bacterial genera. The individual peaks show the magnitude of the influence of each variable on separation of the NC, SC, and BT groups after challenge of broiler chickens (0.05 threshold level)

Of the 212 bacterial OTU identified in this experiment, the 42 differentially abundant bacterial OTU with >1.0% abundance were used to generate a DAPC plot (Fig. 2a) and canonical loading plot (Fig. 2b). The DAPC plot showed that all groups had similar microbial composition before *Salmonella* challenge. But separate clusters were formed in response to *B. subtilis* CSL2 supplementation and/or *S.* Gallinarum infection, suggestive of distinct microbial communities. These microbial shifts were attributed to subtle changes in the abundance of several bacterial OTU, including unclassified *Neisseriaceae*, *Ruminococcus*, and *Candidatus* Arthromitus (Fig. 2b). Unclassified *Neisseriaceae* was the strongest indicator of the presence of distinct microbial clusters; however, the other loading peaks might also exert considerable effects [11].

**Fig. 3:**
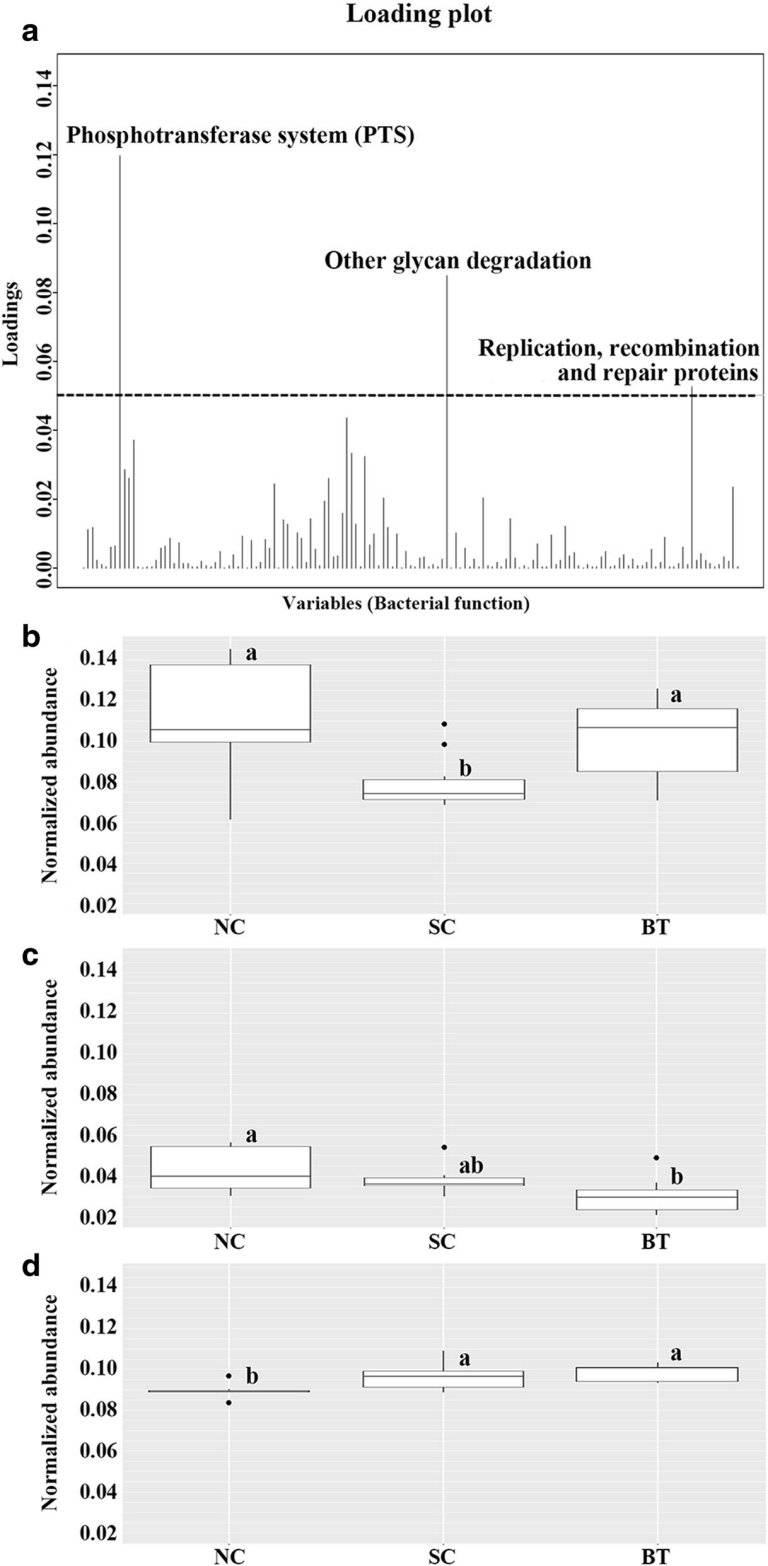
Functional analysis and comparison of microbial communities. **a** Canonical loading plot showing the magnitude of the influence of each variable on separation of individual peaks in the broiler microbiota (0.05 threshold level). Normalised abundances of functional activities **b** phosphotransferase system, **c** glycan degradation, and **d** recombination and repair proteins after *Salmonella* challenge. The interquartile ranges are indicated by the outer bounds of the boxes, the median by the midline (*black*), and the outliers are indicated by black circles (●). Tukey’s honestly significant difference (HSD) test was used to evaluate statistical significance (*P* < 0.05)

Functional analysis identified a total of 137 of 265 KEGG functions as differentially abundant (>0.1% mean relative abundance) (Additional file 4: Table S3). These 137 KEGG functions were analysed using a loading plot to identify vital functions among microbial clusters (Fig. 3a). The three most discriminating KEGG functions in broiler microbiota were the phosphotransferase system (PTS), glycan degradation, and replication/recombination/repair proteins (Fig. 3a). The PTS was significantly decreased by *Salmonella* challenge, but recovered to almost normal levels following *Bacillus* administration (NC group) (Fig. 3b). The abundance of genes associated with glycan degradation was significantly decreased in the BT group compared to the NC and ST groups, which might be a unique effect of supplementation of *B. subtilis* CSL2 (Fig. 3c). In addition, the abundance of genes related to repair and recombination of DNA was highest in the BT group and lowest in the NC group (Fig. 3d).

## Discussion

In this study, *B. subtilis* CSL2 administration altered the microbiota of broilers and contributed to protection against *Salmonella* infection. In previous studies, microbial shifts were evident after probiotic administration and pathogenic invasion [14, 19, 20]. In-feed administration of *E. faecium* NCIMB 11181 changed the microbial composition in piglets [14]. Furthermore, pathogen infection led to significant alterations in the chicken gut microbiota [5, 15, 16]. Pathogen infection disrupts gut microbial diversity and overwhelms the microbiota by producing toxins and harmful agents [15, 18, 19]. Lower microbial diversity indicates a reduced ability of the microbiota to maintain gut homeostasis and resist invasion. *B. subtilis* CSL2 might provide protection against pathogen invasion by increasing bacterial diversity and metabolic and cellular functionality. Reduced functional diversity suppresses commensal-microbiota-mediated homeostasis [21].

Firmicutes and Bacteroidetes comprised the majority of the broiler microbiota at the phylum level; these organisms function in energy production and metabolism, particularly starch digestion and microbial fermentation [4, 10, 13]. The increased abundance of Proteobacteria in *Salmonella*-infected broilers suggests gastrointestinal dysbiosis and imbalance. Indeed, Proteobacteria are closely associated with *S.* Enteritidis infection in animals [15, 16]. Moreover, significant changes were detected in the abundance of *Lactobacillus* and *Turicibacter* after 24 h in the SC and BT groups. *Lactobacillus* is a dominant and important gut commensal genus present at frequencies of up to 10^9^/g [4]. Increased abundance of *Lactobacillus* in the gastrointestinal tract of chickens is considered beneficial for their health and performance [22]. Certain strains of *Lactobacillus* produce antimicrobial substances [23], exopolysaccharides, and short-chain fatty acids as additional energy sources [24]. *B. subtilis* CSL2 promotes the growth of beneficial lactobacilli, as do other types of in-feed probiotics [9, 14].

Furthermore, *Enterobacteriaceae* abundance was reduced in the BT group after 24 h of *Salmonella* challenge, and increased numbers of *Enterobacteriaceae* in SC broilers might imbalance the microbiota and thus exert harmful effects on the gut [20]. The impact of *S.* Gallinarum and *B. subtilis* CSL2 on the overall composition of the gut microbiota can be detected within 24 h post infection [18]. Surprisingly, in this study *Salmonella* spp. were not detected in the SC group. Previous studies also suggest that *Salmonella* abundance is lower after 72 h, during which time symptoms are manifested [15, 19]. Furthermore, the abundance of the genus *Turicibacter* was significantly increased in *Salmonella*-infected broilers. *Turicibacter* has been reported in mammalian studies to be relevant to infection [25]. Its immunomodulatory and invasive properties resulted in subclinical infection of the gastrointestinal tracts of livestock and poultry animals [25, 26]. However, its ecological role and pathogenic potential remain unclear due to the dearth of studies.

A taxon-independent analysis was performed to prevent taxonomic bias and calculate variation based on all differentially abundant bacterial OTU. It is plausible that *B. subtilis* CSL2 maintains microbial community stability, similar to the case in normal broilers. Given that chicken microbial communities are highly dynamic and delicate, abrupt disturbances might cause greater variations in their microbiota than in other animals [4]. In addition, the loading plot of bacterial OTU (i.e. unclassified *Neisseriaceae*, *Ruminococcus*, and *Candidatus* Arthromitus) abundance in the SC group suggested the effects of *S.* Gallinarum infection in broilers. The identification of bacterial OTU belonging to *Neisseriaceae* (phylum: Proteobacteria) was highly suggestive of microbial clustering, and these organisms include several diarrhoea-causing pathogens [27]. These results imply that establishment of *Salmonella* in the gut requires the support of other pathogens or opportunistic bacteria therein.

In this study, functional prediction was performed to evaluate and compare the metabolic activities of the microbial communities among broiler groups [10]. Three metabolic and cellular functions were significantly affected by the administration of *B. subtilis* CSL2 and *S.* Gallinarum infection, namely the phosphotransferase system, glycan degradation, and DNA repair mechanisms. The greater abundance of genes associated with the PTS system in the BT group might be beneficial to broiler chickens. The PTS system is the major bacterial transport system for carbohydrates, and is involved in the regulation of bacterial fermentation and feed conversion [13, 21, 28]. This result thus indicates the probability of rapid uptake of available simple sugars rather than the digestion of complex carbohydrates. Furthermore, Pérez-Cobas et al. suggested that the PTS system assists bacterial stabilisation of gut-associated stresses in an unstable environment, as well as increasing energy yield, giving the commensal microbiota a competitive advantage over foreign microorganisms [29]. In addition, the reduced abundance of genes related to glycan degradation in broilers fed *B. subtilis* CSL2 implies that glycans are not a primary source of nutrients for the microbiota [30]. Higher glycan degradation is significantly correlated with the presence of pathogens (i.e. *Salmonella* and enterotoxigenic Clostridia) [27, 30]. These results suggest that probiotic supplementation alters the metabolic functions of the microbiota in a way that benefits the host. However, additional studies are required to elucidate the protective effects of *B. subtilis* CSL2 against *S.* Gallinarum infection.

## Conclusions

In this study, the characterisation of the chicken faecal microbiota community structure and composition by 16S rRNA gene pyrosequencing revealed that the probiotic strain protected against *Salmonella* infection. The supplementation of *B. subtilis* CSL2 significantly changed the microbial diversity and composition by increasing the abundance of beneficial microorganisms. Conversely, broiler chickens infected with *S.* Gallinarum promoted the growth of potentially harmful bacteria such as *Turicibacter*, *Enterobacteriaceae* and *Neisseriaceae*. Furthermore, the potentially probiotic or pathogenic bacteria influenced the microbial functionality, particularly in the energy transport and metabolism capability of the gut. Overall, *S.* Gallinarum infection and *B. subtilis* CSL2 supplementation in the diet of broiler chickens influenced the diversity, composition, and functional diversity of the faecal microbiota. These results will facilitate prevention of *Salmonella* infection before the onset of symptoms using the potentially probiotic strain *B. subtilis* CSL2. Moreover, the findings offer significant insights to understand potential mechanisms of *Salmonella* infection and the mode of action of probiotics in broiler chickens.

## Additional files

Additional file 1: Table S1. Basal diet composition. (DOCX 19 kb)
Additional file 2: Figure S1. Rarefaction curves measuring bacterial diversity among broiler communities. (DOCX 231 kb)
Additional file 3: Table S2. Highly abundant genera in broiler groups. (DOCX 22 kb)
Additional file 4: Table S3. Highly abundant KEGG functions in broiler groups. (DOCX 22 kb)

## Abbreviations

BT: *Bacillus subtilis* CSL2-treated
DAPC: Discriminant analysis of principal components
KEGG: Kyoto encyclopedia of genes and genomes
NC: Negative control
NGS: Next generation sequencing
OTU: Operational taxonomic unit
PICRUSt: Phylogenetic investigation of communities by reconstruction of unobserved states
QIIME: Quantitative insights into microbial ecology
SC: *Salmonella*-challenged/*Salmonella*-infected chickens
