# Supplementary material for: Effects of Bacillus subtilis CSL2 on the composition and functional diversity of the faecal microbiota of broiler chickens challenged with Salmonella Gallinarum
Source: J Anim Sci Biotechnol. 2017 Jan 5;8:1. doi: 10.1186/s40104-016-0130-8 (PMC5215103; doi:10.1186/s40104-016-0130-8)
Supplement: Additional file 1: Table S1. — Basal diet composition. (DOCX 19 kb) [file 40104_2016_130_MOESM1_ESM.docx]

**Table S1** Basal diet composition (as-fed basis)

| **Item** | | **Starter^1^** | **Grower** |
| --- | --- | --- | --- |
| **Ingredients (%)** | |  |  |
| Corn | 55.42 | 62.98 | |
| Soybean meal (CP 48%) | 28.25 | 24.61 | |
| Corn gluten meal (CP 60%) | 6.50 | 3.50 | |
| Soybean oil | 5.50 | 4.89 | |
| Dicalcium phosphate | 2.46 | 2.29 | |
| Limestone | 0.89 | 0.75 | |
| Salt | 0.20 | 0.20 | |
| DL-Methionine (98%) | 0.17 | 0.17 | |
| L-Lysine-HCl (78%) | 0.21 | 0.21 | |
| Vitamin premix^2^ | 0.20 | 0.20 | |
| Mineral premix^3^ | 0.20 | 0.20 | |
| Total | 100.00 | 100.00 | |
| **Chemical composition** | |  |  |
| Metabolizable energy (ME) (kcal/kg) | 3,140 | 3,200 | |
| Crude Protein (CP) (%) | 22.00 | 20.09 | |
| Lysine (%) | 1.10 | 1.05 | |
| Met (%) | 0.54 | 0.41 | |
| Met + Cys (%) | 0.93 | 0.93 | |
| Ca (%) | 1.00 | 0.87 | |
| Total P (%) | 0.50 | 0.75 | |
| Crude Fat (%) | 4.32 | 5.87 | |
| Crude Fiber (%) | 4.71 | 6.21 | |

^1^Starter diets provided during d 1 to 18; grower diet provided during d 19 to 33.

^2^Provided per kg of complete diet: 15,000 IU of vitamin A, 3,750 IU of vitamin D_3_, 37.5 mg of vitamin E, 2.55 mg of vitamin K_3_, 3 mg of thiamin, 7.5 mg of riboflavin, 4.5 mg of vitamin B_6_, 24 ug of vitamin B_12_, 51 mg of niacin, 1.5 mg of folic acid, 0.2 mg of biotin and 13.5 mg of Ca-Pantothenate.

^3^Provided per kg of complete diet: 37.5 mg Zn (as ZnSO_4_); 37.5 mg Mn (as MnO_2_); 37.5 mg Fe (as FeSO_4_∙7H_2_O); 3.75 mg Cu (as CuSO_4_∙5H_2_O); 0.83 mg I (as KI); and 0.23 mg Se (as Na_2_SeO_3_∙5H_2_O)
