# Supplementary material for: Effects of Bacillus subtilis CSL2 on the composition and functional diversity of the faecal microbiota of broiler chickens challenged with Salmonella Gallinarum
Source: J Anim Sci Biotechnol. 2017 Jan 5;8:1. doi: 10.1186/s40104-016-0130-8 (PMC5215103; doi:10.1186/s40104-016-0130-8)
Supplement: Additional file 2: Figure S1. — Rarefaction curves measuring bacterial diversity among broiler communities. (DOCX 231 kb) [file 40104_2016_130_MOESM2_ESM.docx]

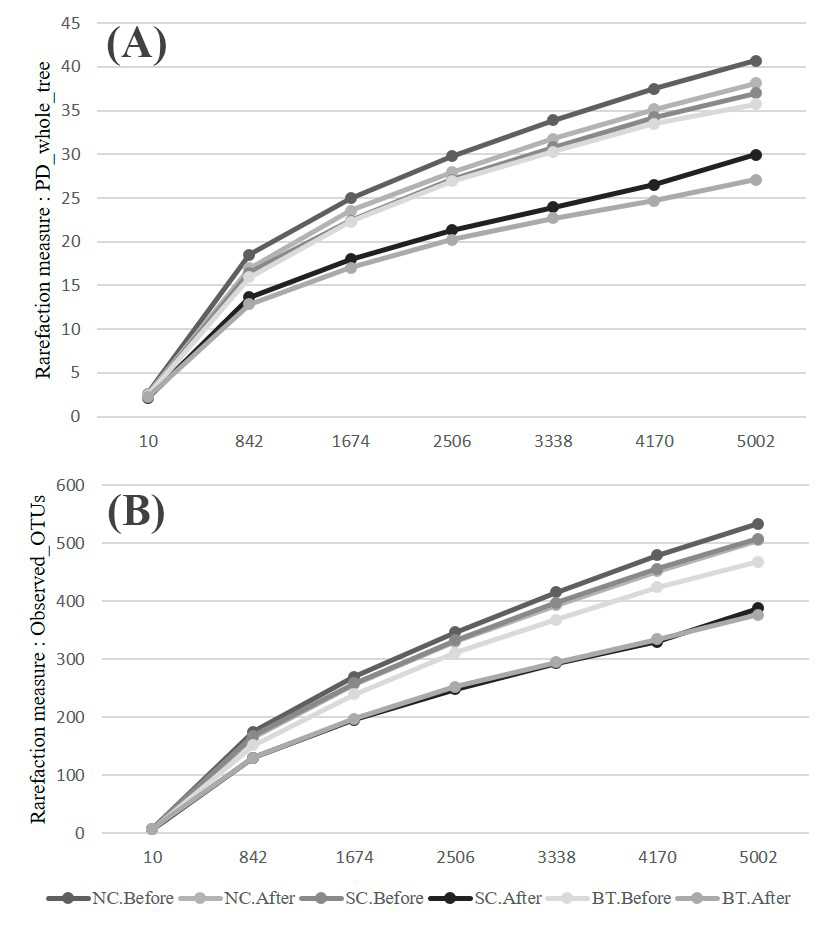


**Fig. S1** Rarefaction curves measuring bacterial diversity among broiler communities. Alpha-diversity measured by (A) PD_whole_tree and (B) Observed OTUs before and after *Salmonella* infection in control (NC), *Salmonella*-challenged (SC), and *Bacillus*-treated (BT) groups.
