# Supplementary material for: Effects of Bacillus subtilis CSL2 on the composition and functional diversity of the faecal microbiota of broiler chickens challenged with Salmonella Gallinarum
Source: J Anim Sci Biotechnol. 2017 Jan 5;8:1. doi: 10.1186/s40104-016-0130-8 (PMC5215103; doi:10.1186/s40104-016-0130-8)
Supplement: Additional file 3: Table S2. — Highly abundant genera in broiler groups. (DOCX 22 kb) [file 40104_2016_130_MOESM3_ESM.docx]

**Table S2** Bacterial genera (n = 124) present in broiler chickens

| Genus (#1~31) | Genus (#32~63) | Genus (#64~95) | Genus (#96~124) |
| --- | --- | --- | --- |
| *Lactobacillus* | *f_Erysipelotrichaceae;g_cc_115* | *Zoogloea* | *Arcobacter* |
| *Turicibacter* | *Chryseobacterium* | *Anaerococcus* | *Rubellimicrobium* |
| *Bacteroides* | *Pseudoramibacter_Eubacterium* | *Wautersiella* | *Burkholderia* |
| *Enterococcus* | *Anaeroplasma* | *Bilophila* | *Pedobacter* |
| *Candidatus Arthromitus* | *Anaerostipes* | *Aerococcus* | *Rothia* |
| *Faecalibacterium* | *Facklamia* | *Micrococcus* | *Trabulsiella* |
| *Oscillospira* | *Fusobacterium* | *Lysobacter* | *Legionella* |
| *Sutterella* | *Roseburia* | *Actinomycetospora* | *Fimbriimonas* |
| *f_Clostridiaceae;g_Clostridium* | *Coprobacillus* | *Nevskia* | *Vagococcus* |
| *f_Lachnospiraceae;g_[Ruminococcus]* | *Proteiniclasticum* | *Myroides* | *Gelidibacter* |
| *f_Ruminococcaceae;g_Ruminococcus* | *Sphingobacterium* | *Psychrobacter* | *Methyloversatilis* |
| *Helicobacter* | *Jeotgalicoccus* | *Luteimonas* | *Methylopila* |
| *f_Clostridiaceae;g_SMB53* | *Gordonia* | *Carnobacterium* | *Trichococcus* |
| *Coprococcus* | *Phascolarctobacterium* | *Planomicrobium* | *Leucobacter* |
| *Blautia* | *Enhydrobacter* | *Butyricicoccus* | *Haemophilus* |
| *Acinetobacter* | *Succinatimonas* | *Lysinibacillus* | *Wohlfahrtiimonas* |
| *Epulopiscium* | *Sphingomonas* | *Serratia* | *Kaistobacter* |
| *Corynebacterium* | *Gallicola* | *Lachnospira* | *Capnocytophaga* |
| *f_Lachnospiraceae;g_Clostridium* | *Peptococcus* | *Knoellia* | *Tsukamurella* |
| *f_Erysipelotrichaceae;g_[Eubacterium]* | *Dehalobacterium* | *Lautropia* | *Oligella* |
| *Proteus* | *Streptococcus* | *f_Clostridiaceae;g_02d06* | *Solibacillus* |
| *Dorea* | *Mucispirillum* | *Rhodococcus* | *Akkermansia* |
| *Odoribacter* | *Brachybacterium* | *Mycobacterium* | *Methylobacterium* |
| *Staphylococcus* | *o_Bacteroidales;f_[Paraprevotellaceae];g_[Prevotella]* | *Planococcus* | *Paracoccus* |
| *Anaerotruncus* | *Comamonas* | *Holdemania* | *Saccharopolyspora* |
| *Pseudomonas* | *Brevibacterium* | *Sporosarcina* | *Eggerthella* |
| *Arthrobacter* | *Truepera* | *Kocuria* | *Janibacter* |
| *Parabacteroides* | *f_Peptostreptococcaceae;g_Clostridium* | *Aequorivita* | *Caloramator* |
| *Prevotella* | *Finegoldia* | *Sphingobium* | *Actinomyces* |
| *Flexispira* | *Anaerofustis* | *Ochrobactrum* | *Campylobacter* |
| *Bacillus* | *Dysgonomonas* | *Delftia* | *Novosphingobium* |
