# Supplementary material for: Effects of Bacillus subtilis CSL2 on the composition and functional diversity of the faecal microbiota of broiler chickens challenged with Salmonella Gallinarum
Source: J Anim Sci Biotechnol. 2017 Jan 5;8:1. doi: 10.1186/s40104-016-0130-8 (PMC5215103; doi:10.1186/s40104-016-0130-8)
Supplement: Additional file 4: Table S3. — Highly abundant KEGG functions in broiler groups. (DOCX 22 kb) [file 40104_2016_130_MOESM4_ESM.docx]

**Table S3** Highly abundant KEGG functions in broiler groups (> 0.1% mean relative abundance)

| Function (#1~35) | Function (#36~70) | Function (#71~105) | Function (#106~137) |
| --- | --- | --- | --- |
| Transporters | Alanine, aspartate and glutamate metabolism | Pentose and glucuronate interconversions | C5-Branched dibasic acid metabolism |
| General function prediction only | Cysteine and methionine metabolism | Glycerolipid metabolism | Aminobenzoate degradation |
| ABC transporters | Mismatch repair | RNA degradation | Cell motility and secretion |
| DNA repair and recombination proteins | Pentose phosphate pathway | Flagellar assembly | Tetracycline biosynthesis |
| Ribosome | Peptidoglycan biosynthesis | Tyrosine metabolism | RNA transport |
| Purine metabolism | Energy metabolism | Glyoxylate and dicarboxylate metabolism | Inorganic ion transport and metabolism |
| Peptidases | Galactose metabolism | Nucleotide excision repair | D-Glutamine and D-glutamate metabolism |
| Pyrimidine metabolism | Butanoate metabolism | Cytoskeleton proteins | Plant-pathogen interaction |
| Transcription factors | DNA replication | Nicotinate and nicotinamide metabolism | Tuberculosis |
| Chromosome | Lysine biosynthesis | Membrane and intracellular structural molecules | D-Alanine metabolism |
| Two-component system | Glycine, serine and threonine metabolism | Photosynthesis proteins | Tryptophan metabolism |
| Amino sugar and nucleotide sugar metabolism | Sporulation | Glycosyltransferases | Other glycan degradation |
| Ribosome Biogenesis | Porphyrin and chlorophyll metabolism | Photosynthesis | Metabolism of cofactors and vitamins |
| Other ion-coupled transporters | Terpenoid backbone biosynthesis | Selenocompound metabolism | Polyketide sugar unit biosynthesis |
| Amino acid related enzymes | Nitrogen metabolism | Drug metabolism - other enzymes | Phenylalanine metabolism |
| Function unknown | Carbon fixation in photosynthetic organisms | Prenyltransferases | Bacterial toxins |
| DNA replication proteins | Protein export | Benzoate degradation | Lipid metabolism |
| Secretion system | Glycerophospholipid metabolism | Folate biosynthesis | Lysine degradation |
| Aminoacyl-tRNA biosynthesis | One carbon pool by folate | Sulfur relay system | Ubiquinone and other terpenoid-quinone biosynthesis |
| Glycolysis / Gluconeogenesis | Translation factors | Riboflavin metabolism | Carbohydrate metabolism |
| Metabolism;Others | Propanoate metabolism | Fatty acid metabolism | Taurine and hypotaurine metabolism |
| Fructose and mannose metabolism | Bacterial secretion system | Protein kinases | beta-Alanine metabolism |
| Methane metabolism | Valine, leucine and isoleucine biosynthesis | Glutathione metabolism | Vitamin B6 metabolism |
| Pyruvate metabolism | Signal transduction mechanisms | Streptomycin biosynthesis | Peroxisome |
| Arginine and proline metabolism | Citrate cycle (TCA cycle) | Cyanoamino acid metabolism | Sphingolipid metabolism |
| Homologous recombination | Lipid biosynthesis proteins | Valine, leucine and isoleucine degradation | Limonene and pinene degradation |
| Starch and sucrose metabolism | Phenylalanine, tyrosine and tryptophan biosynthesis | Pores ion channels | Biosynthesis of unsaturated fatty acids |
| Phosphotransferase system (PTS) | Protein folding and associated processing | Cellular Processes and Signaling;Other transporters | Polycyclic aromatic hydrocarbon degradation |
| Oxidative phosphorylation | Cell cycle - Caulobacter | Amino acid metabolism | Toluene degradation |
| Replication, recombination and repair proteins | Pantothenate and CoA biosynthesis | Chloroalkane and chloroalkene degradation | Novobiocin biosynthesis |
| Transcription machinery | Fatty acid biosynthesis | Restriction enzyme | Lipopolysaccharide biosynthesis |
| Translation proteins | Thiamine metabolism | Sulfur metabolism | Tropane, piperidine and pyridine alkaloid biosynthesis |
| Chaperones and folding catalysts | Base excision repair | Naphthalene degradation |  |
| Carbon fixation pathways in prokaryotes | Histidine metabolism | Lipopolysaccharide biosynthesis proteins |  |
| Bacterial motility proteins | Bacterial chemotaxis | RNA polymerase |  |
